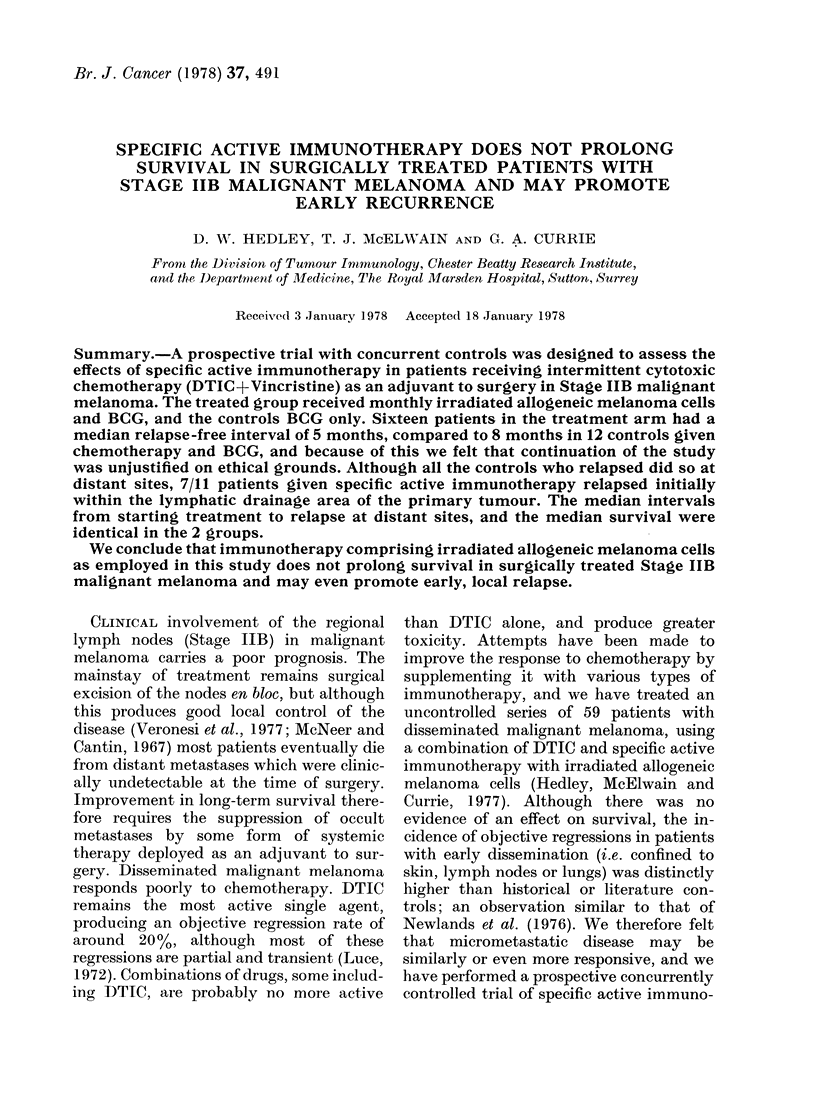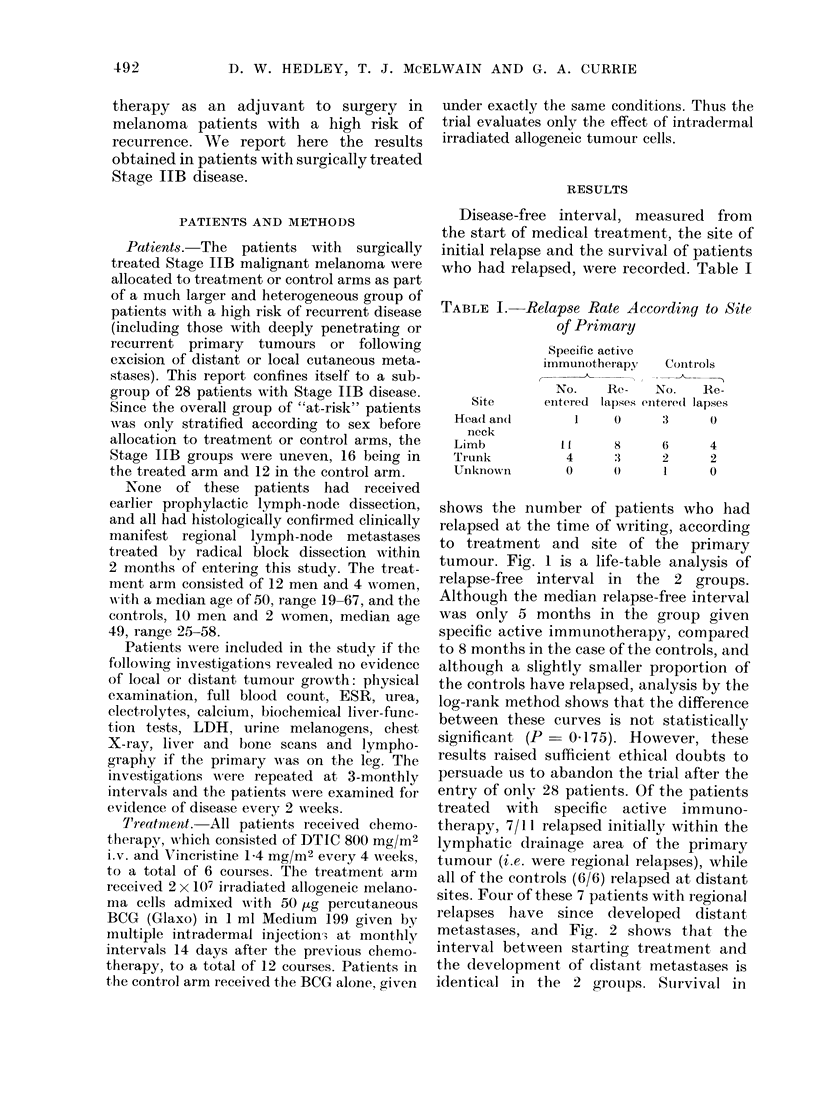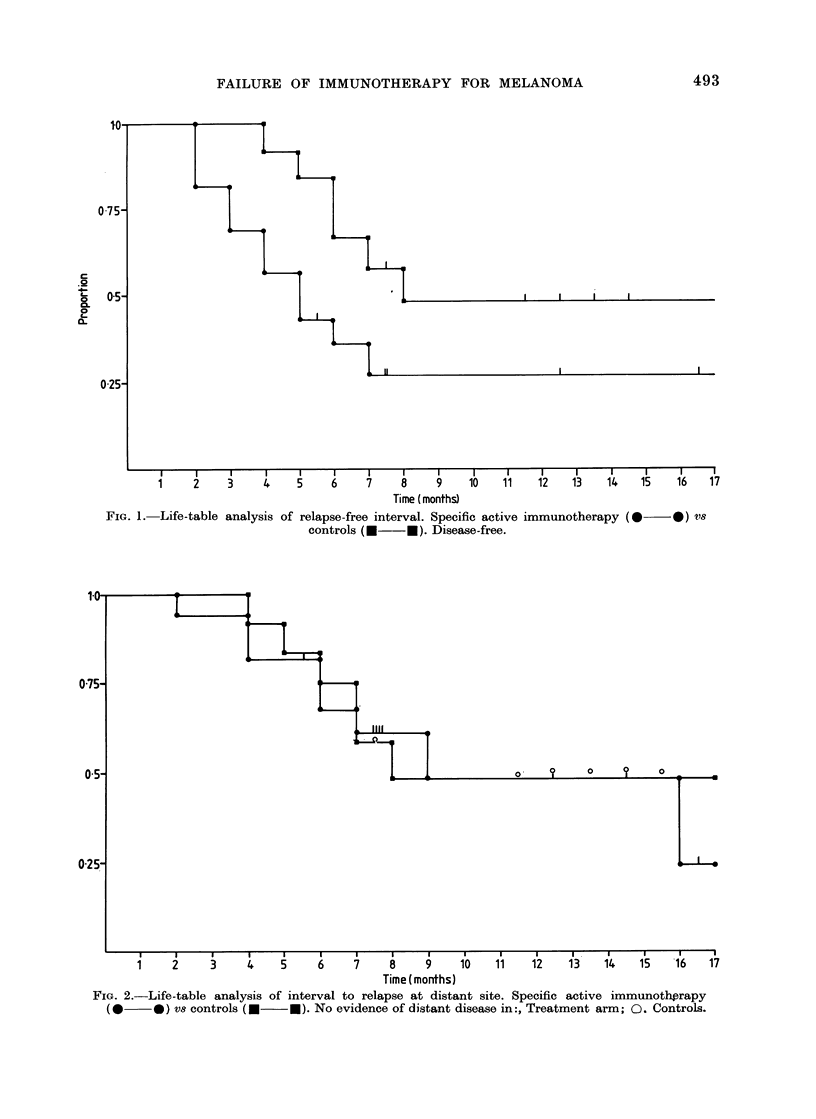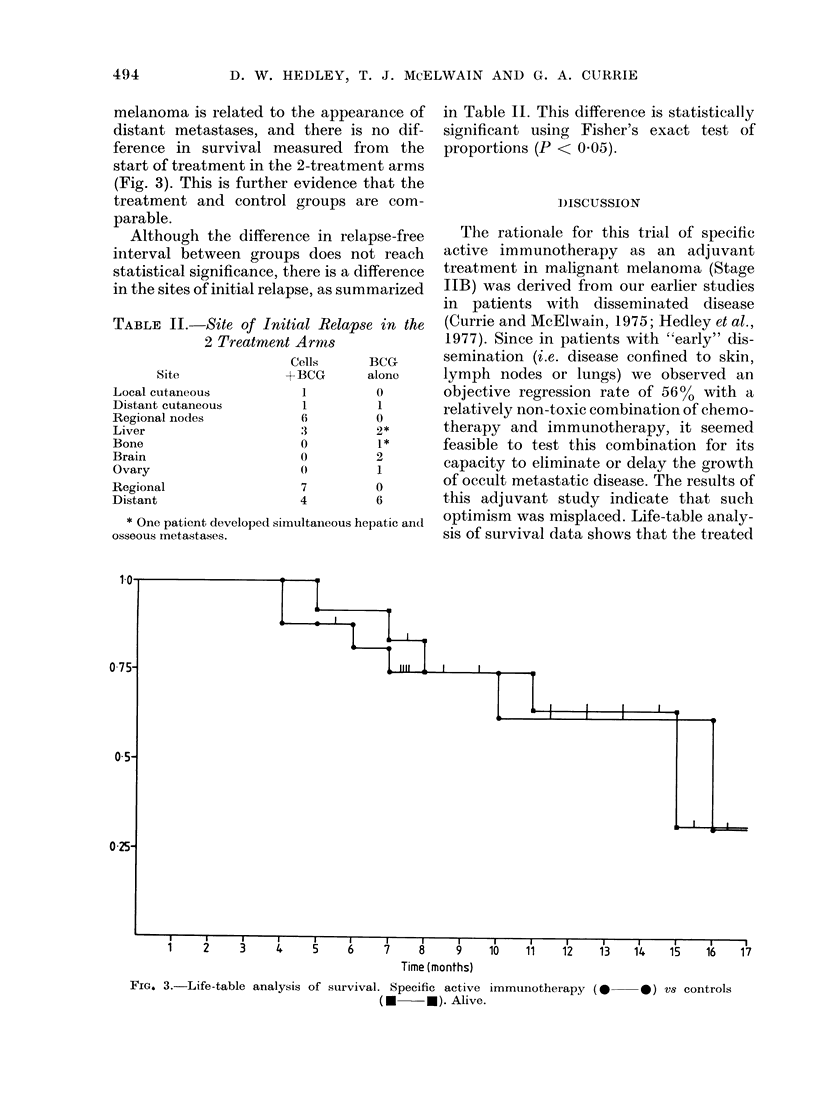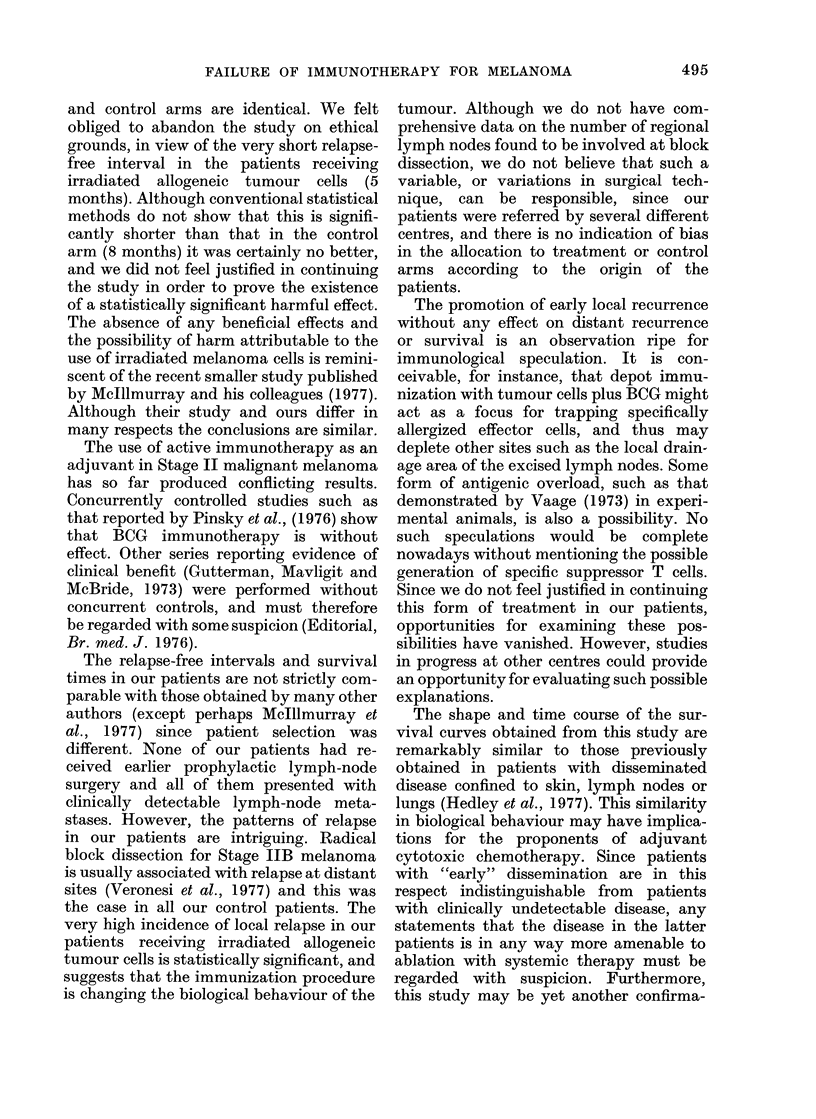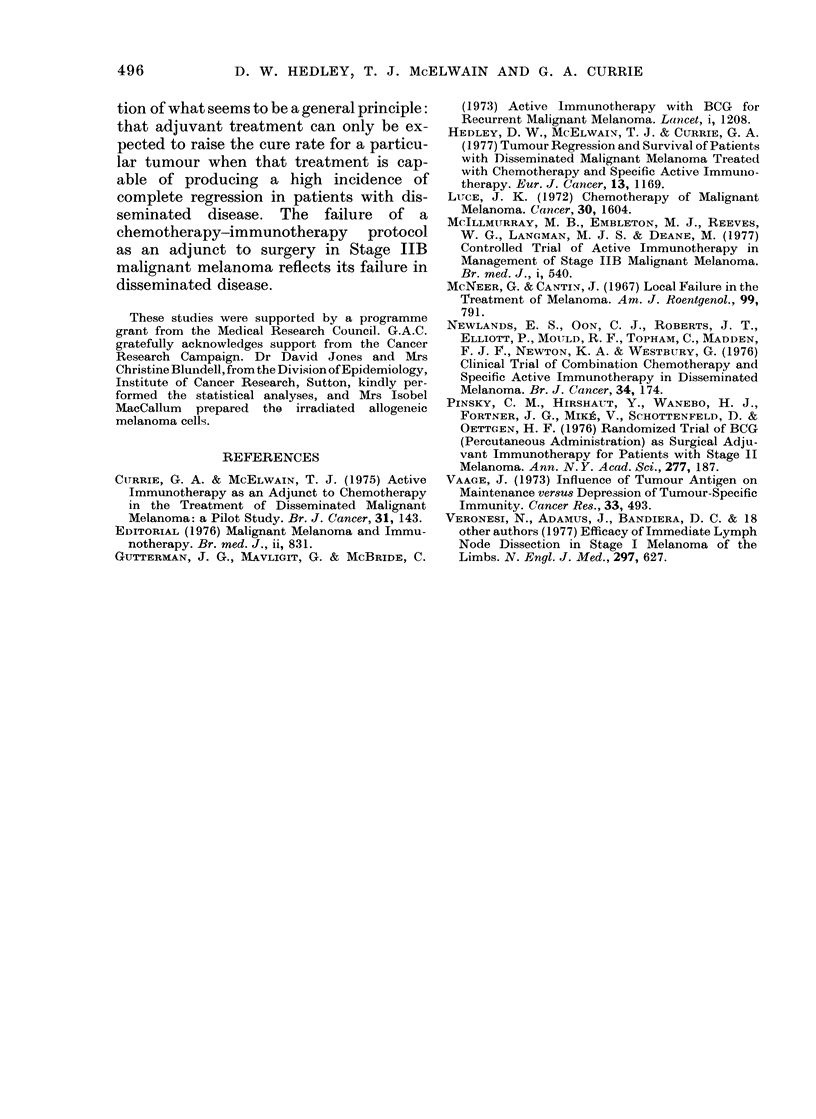# Specific active immunotherapy does not prolong survival in surgically treated patients with stage IIB malignant melanoma and may promote early recurrence.

**DOI:** 10.1038/bjc.1978.76

**Published:** 1978-04

**Authors:** D. W. Hedley, T. J. McElwain, G. A. Currie

## Abstract

A prospective trial with concurrent controls was designed to assess the effects of specific active immunotherapy in patients receiving intermittent cytotoxic chemotherapy (DTIC + Vincristine) as an adjuvant to surgery in Stage IIB malignant melanoma. The treated group received monthly irradiated allogeneic melanoma cells and BCG, and the controls BCG only. Sixteen patients in the treatment arm had a median relapse-free interval of 5 months, compared to 8 months in 12 controls given chemotherapy and BCG, and because of this we felt that continuation of the study was unjustified on ethical grounds. Although all the controls who relapsed did so at distant sites, 7/11 patients given specific active immunotherapy relapsed initially within the lymphatic drainage area of the primary tumour. The median intervals from starting treatment to relapse at distant sites, and the median survival were identical in the 2 groups. We conclude that immunotherapy comprising irradiated allogenic melanoma cells as employed in this study does not prolong survival in surgically treated Stage IIB malignant melanoma and may even promote early, local relapse.


*Br. J. Cancer* (1978) **37**, 491

### SPECIFIC ACTIVE IMMUNOTHERAPY DOES NOT PROLONG SURVIVAL IN SURGICALLY TREATED PATIENTS WITH STAGE IIB MALIGNANT MELANOMA AND MAY PROMOTE EARLY RECURRENCE

D. W. HEDLEY, T. J. McELWAIN AND G. A. CURRIE

*From the Division of Tumour Immunology, Chester Beatty Research Institute, and the Department of Medicine, The Royal Marsden Hospital, Sutton, Surrey*

Received 3 January 1978  Accepted 18 January 1978

**Summary.—A prospective trial with concurrent controls was designed to assess the effects of specific active immunotherapy in patients receiving intermittent cytotoxic chemotherapy (DTIC+Vincristine) as an adjuvant to surgery in Stage IIB malignant melanoma. The treated group received monthly irradiated allogeneic melanoma cells and BCG, and the controls BCG only. Sixteen patients in the treatment arm had a median relapse-free interval of 5 months, compared to 8 months in 12 controls given chemotherapy and BCG, and because of this we felt that continuation of the study was unjustified on ethical grounds. Although all the controls who relapsed did so at distant sites, 7/11 patients given specific active immunotherapy relapsed initially within the lymphatic drainage area of the primary tumour. The median intervals from starting treatment to relapse at distant sites, and the median survival were identical in the 2 groups.**

**We conclude that immunotherapy comprising irradiated allogeneic melanoma cells as employed in this study does not prolong survival in surgically treated Stage IIB malignant melanoma and may even promote early, local relapse.**

CLINICAL involvement of the regional lymph nodes (Stage IIB) in malignant melanoma carries a poor prognosis. The mainstay of treatment remains surgical excision of the nodes *en bloc*, but although this produces good local control of the disease (Veronesi *et al.*, 1977; McNeer and Cantin, 1967) most patients eventually die from distant metastases which were clinically undetectable at the time of surgery. Improvement in long-term survival therefore requires the suppression of occult metastases by some form of systemic therapy deployed as an adjuvant to surgery. Disseminated malignant melanoma responds poorly to chemotherapy. DTIC remains the most active single agent, producing an objective regression rate of around 20%, although most of these regressions are partial and transient (Luce, 1972). Combinations of drugs, some including DTIC, are probably no more active than DTIC alone, and produce greater toxicity. Attempts have been made to improve the response to chemotherapy by supplementing it with various types of immunotherapy, and we have treated an uncontrolled series of 59 patients with disseminated malignant melanoma, using a combination of DTIC and specific active immunotherapy with irradiated allogeneic melanoma cells (Hedley, McElwain and Currie, 1977). Although there was no evidence of an effect on survival, the incidence of objective regressions in patients with early dissemination (*i.e.* confined to skin, lymph nodes or lungs) was distinctly higher than historical or literature controls; an observation similar to that of Newlands *et al.* (1976). We therefore felt that micrometastatic disease may be similarly or even more responsive, and we have performed a prospective concurrently controlled trial of specific active immunotherapy as an adjuvant to surgery in melanoma patients with a high risk of recurrence. We report here the results obtained in patients with surgically treated Stage IIB disease.

### PATIENTS AND METHODS

*Patients.*—The patients with surgically treated Stage IIB malignant melanoma were allocated to treatment or control arms as part of a much larger and heterogeneous group of patients with a high risk of recurrent disease (including those with deeply penetrating or recurrent primary tumours or following excision of distant or local cutaneous metastases). This report confines itself to a subgroup of 28 patients with Stage IIB disease. Since the overall group of "at-risk" patients was only stratified according to sex before allocation to treatment or control arms, the Stage IIB groups were uneven, 16 being in the treated arm and 12 in the control arm.

None of these patients had received earlier prophylactic lymph-node dissection, and all had histologically confirmed clinically manifest regional lymph-node metastases treated by radical block dissection within 2 months of entering this study. The treatment arm consisted of 12 men and 4 women, with a median age of 50, range 19–67, and the controls, 10 men and 2 women, median age 49, range 25–58.

Patients were included in the study if the following investigations revealed no evidence of local or distant tumour growth: physical examination, full blood count, ESR, urea, electrolytes, calcium, biochemical liver-function tests, LDH, urine melanogens, chest X-ray, liver and bone scans and lymphography if the primary was on the leg. The investigations were repeated at 3-monthly intervals and the patients were examined for evidence of disease every 2 weeks.

*Treatment.*—All patients received chemotherapy, which consisted of DTIC 800 mg/m$^2$ i.v. and Vincristine 1·4 mg/m$^2$ every 4 weeks, to a total of 6 courses. The treatment arm received $2 \times 10^7$ irradiated allogeneic melanoma cells admixed with 50 $\mu$g percutaneous BCG (Glaxo) in 1 ml Medium 199 given by multiple intradermal injections at monthly intervals 14 days after the previous chemotherapy, to a total of 12 courses. Patients in the control arm received the BCG alone, given under exactly the same conditions. Thus the trial evaluates only the effect of intradermal irradiated allogeneic tumour cells.

### RESULTS

Disease-free interval, measured from the start of medical treatment, the site of initial relapse and the survival of patients who had relapsed, were recorded. Table I

TABLE I.—*Relapse Rate According to Site of Primary*

| | Specific active immunotherapy | | Controls | |
|---|---|---|---|---|
| Site | No. entered | Relapses | No. entered | Relapses |
| Head and neck | 1 | 0 | 3 | 0 |
| Limb | 11 | 8 | 6 | 4 |
| Trunk | 4 | 3 | 2 | 2 |
| Unknown | 0 | 0 | 1 | 0 |

shows the number of patients who had relapsed at the time of writing, according to treatment and site of the primary tumour. Fig. 1 is a life-table analysis of relapse-free interval in the 2 groups. Although the median relapse-free interval was only 5 months in the group given specific active immunotherapy, compared to 8 months in the case of the controls, and although a slightly smaller proportion of the controls have relapsed, analysis by the log-rank method shows that the difference between these curves is not statistically significant ($P = 0{\cdot}175$). However, these results raised sufficient ethical doubts to persuade us to abandon the trial after the entry of only 28 patients. Of the patients treated with specific active immunotherapy, 7/11 relapsed initially within the lymphatic drainage area of the primary tumour (*i.e.* were regional relapses), while all of the controls (6/6) relapsed at distant sites. Four of these 7 patients with regional relapses have since developed distant metastases, and Fig. 2 shows that the interval between starting treatment and the development of distant metastases is identical in the 2 groups. Survival in

FIG. 1.—Life-table analysis of relapse-free interval. Specific active immunotherapy (●——●) *vs* controls (■——■). Disease-free.

FIG. 2.—Life-table analysis of interval to relapse at distant site. Specific active immunotherapy (●——●) *vs* controls (■——■). No evidence of distant disease in:, Treatment arm; ○. Controls.

melanoma is related to the appearance of distant metastases, and there is no difference in survival measured from the start of treatment in the 2-treatment arms (Fig. 3). This is further evidence that the treatment and control groups are comparable.

Although the difference in relapse-free interval between groups does not reach statistical significance, there is a difference in the sites of initial relapse, as summarized in Table II. This difference is statistically significant using Fisher's exact test of proportions ($P < 0{\cdot}05$).

TABLE II.—*Site of Initial Relapse in the 2 Treatment Arms*

| Site | Cells +BCG | BCG alone |
|---|---|---|
| Local cutaneous | 1 | 0 |
| Distant cutaneous | 1 | 1 |
| Regional nodes | 6 | 0 |
| Liver | 3 | 2* |
| Bone | 0 | 1* |
| Brain | 0 | 2 |
| Ovary | 0 | 1 |
| Regional | 7 | 0 |
| Distant | 4 | 6 |

* One patient developed simultaneous hepatic and osseous metastases.

### DISCUSSION

The rationale for this trial of specific active immunotherapy as an adjuvant treatment in malignant melanoma (Stage IIB) was derived from our earlier studies in patients with disseminated disease (Currie and McElwain, 1975; Hedley *et al.*, 1977). Since in patients with "early" dissemination (*i.e.* disease confined to skin, lymph nodes or lungs) we observed an objective regression rate of 56% with a relatively non-toxic combination of chemotherapy and immunotherapy, it seemed feasible to test this combination for its capacity to eliminate or delay the growth of occult metastatic disease. The results of this adjuvant study indicate that such optimism was misplaced. Life-table analysis of survival data shows that the treated

FIG. 3.—Life-table analysis of survival. Specific active immunotherapy (●——●) *vs* controls (■——■). Alive.

and control arms are identical. We felt obliged to abandon the study on ethical grounds, in view of the very short relapse-free interval in the patients receiving irradiated allogeneic tumour cells (5 months). Although conventional statistical methods do not show that this is significantly shorter than that in the control arm (8 months) it was certainly no better, and we did not feel justified in continuing the study in order to prove the existence of a statistically significant harmful effect. The absence of any beneficial effects and the possibility of harm attributable to the use of irradiated melanoma cells is reminiscent of the recent smaller study published by McIllmurray and his colleagues (1977). Although their study and ours differ in many respects the conclusions are similar.

The use of active immunotherapy as an adjuvant in Stage II malignant melanoma has so far produced conflicting results. Concurrently controlled studies such as that reported by Pinsky *et al.*, (1976) show that BCG immunotherapy is without effect. Other series reporting evidence of clinical benefit (Gutterman, Mavligit and McBride, 1973) were performed without concurrent controls, and must therefore be regarded with some suspicion (Editorial, *Br. med. J.* 1976).

The relapse-free intervals and survival times in our patients are not strictly comparable with those obtained by many other authors (except perhaps McIllmurray *et al.*, 1977) since patient selection was different. None of our patients had received earlier prophylactic lymph-node surgery and all of them presented with clinically detectable lymph-node metastases. However, the patterns of relapse in our patients are intriguing. Radical block dissection for Stage IIB melanoma is usually associated with relapse at distant sites (Veronesi *et al.*, 1977) and this was the case in all our control patients. The very high incidence of local relapse in our patients receiving irradiated allogeneic tumour cells is statistically significant, and suggests that the immunization procedure is changing the biological behaviour of the tumour. Although we do not have comprehensive data on the number of regional lymph nodes found to be involved at block dissection, we do not believe that such a variable, or variations in surgical technique, can be responsible, since our patients were referred by several different centres, and there is no indication of bias in the allocation to treatment or control arms according to the origin of the patients.

The promotion of early local recurrence without any effect on distant recurrence or survival is an observation ripe for immunological speculation. It is conceivable, for instance, that depot immunization with tumour cells plus BCG might act as a focus for trapping specifically allergized effector cells, and thus may deplete other sites such as the local drainage area of the excised lymph nodes. Some form of antigenic overload, such as that demonstrated by Vaage (1973) in experimental animals, is also a possibility. No such speculations would be complete nowadays without mentioning the possible generation of specific suppressor T cells. Since we do not feel justified in continuing this form of treatment in our patients, opportunities for examining these possibilities have vanished. However, studies in progress at other centres could provide an opportunity for evaluating such possible explanations.

The shape and time course of the survival curves obtained from this study are remarkably similar to those previously obtained in patients with disseminated disease confined to skin, lymph nodes or lungs (Hedley *et al.*, 1977). This similarity in biological behaviour may have implications for the proponents of adjuvant cytotoxic chemotherapy. Since patients with "early" dissemination are in this respect indistinguishable from patients with clinically undetectable disease, any statements that the disease in the latter patients is in any way more amenable to ablation with systemic therapy must be regarded with suspicion. Furthermore, this study may be yet another confirmation of what seems to be a general principle: that adjuvant treatment can only be expected to raise the cure rate for a particular tumour when that treatment is capable of producing a high incidence of complete regression in patients with disseminated disease. The failure of a chemotherapy–immunotherapy protocol as an adjunct to surgery in Stage IIB malignant melanoma reflects its failure in disseminated disease.

These studies were supported by a programme grant from the Medical Research Council. G.A.C. gratefully acknowledges support from the Cancer Research Campaign. Dr David Jones and Mrs Christine Blundell, from the Division of Epidemiology, Institute of Cancer Research, Sutton, kindly performed the statistical analyses, and Mrs Isobel MacCallum prepared the irradiated allogeneic melanoma cells.

### REFERENCES

CURRIE, G. A. & McELWAIN, T. J. (1975) Active Immunotherapy as an Adjunct to Chemotherapy in the Treatment of Disseminated Malignant Melanoma: a Pilot Study. *Br. J. Cancer*, **31**, 143.

EDITORIAL (1976) Malignant Melanoma and Immunotherapy. *Br. med. J.*, ii, 831.

GUTTERMAN, J. G., MAVLIGIT, G. & McBRIDE, C. (1973) Active Immunotherapy with BCG for Recurrent Malignant Melanoma. *Lancet*, i, 1208.

HEDLEY, D. W., McELWAIN, T. J. & CURRIE, G. A. (1977) Tumour Regression and Survival of Patients with Disseminated Malignant Melanoma Treated with Chemotherapy and Specific Active Immunotherapy. *Eur. J. Cancer*, **13**, 1169.

LUCE, J. K. (1972) Chemotherapy of Malignant Melanoma. *Cancer*, **30**, 1604.

McILLMURRAY, M. B., EMBLETON, M. J., REEVES, W. G., LANGMAN, M. J. S. & DEANE, M. (1977) Controlled Trial of Active Immunotherapy in Management of Stage IIB Malignant Melanoma. *Br. med. J.*, i, 540.

McNEER, G. & CANTIN, J. (1967) Local Failure in the Treatment of Melanoma. *Am. J. Roentgenol.*, **99**, 791.

NEWLANDS, E. S., OON, C. J., ROBERTS, J. T., ELLIOTT, P., MOULD, R. F., TOPHAM, C., MADDEN, F. J. F., NEWTON, K. A. & WESTBURY, G. (1976) Clinical Trial of Combination Chemotherapy and Specific Active Immunotherapy in Disseminated Melanoma. *Br. J. Cancer*, **34**, 174.

PINSKY, C. M., HIRSHAUT, Y., WANEBO, H. J., FORTNER, J. G., MIKÉ, V., SCHOTTENFELD, D. & OETTGEN, H. F. (1976) Randomized Trial of BCG (Percutaneous Administration) as Surgical Adjuvant Immunotherapy for Patients with Stage II Melanoma. *Ann. N.Y. Acad. Sci.*, **277**, 187.

VAAGE, J. (1973) Influence of Tumour Antigen on Maintenance *versus* Depression of Tumour-Specific Immunity. *Cancer Res.*, **33**, 493.

VERONESI, N., ADAMUS, J., BANDIERA, D. C. & 18 other authors (1977) Efficacy of Immediate Lymph Node Dissection in Stage I Melanoma of the Limbs. *N. Engl. J. Med.*, **297**, 627.